# Integration of Large Language Models in Personal Statements for Neurosurgical Residency Applications: Insights from a Student Survey

**DOI:** 10.7759/cureus.99409

**Published:** 2025-12-16

**Authors:** Cameron A Rivera, Vratko Himic, Nathan Zwagerman, Ashish H Shah, Michael Ivan, Ricardo J Komotar, Daniel M Aaronson

**Affiliations:** 1 Neurological Surgery, University of Miami Miller School of Medicine, Miami, USA; 2 Neurosurgery, Medical College of Wisconsin, Milwaukee, USA

**Keywords:** artificial intelligence, large language model, neurosurgery, personal statement, residency

## Abstract

Large language models (LLMs) are presumed to play an increasing role in residency applications. Given the role of personal statements in the holistic review of neurosurgical residency applicants, the extent of LLM influence remains unexplored. A multicenter survey was administered to residency interviewees during the 2024-2025 interview cycle. Respondents anonymously reported their use of LLM tools while writing personal statements. A total of 35 survey responses were recorded. All results were anonymized to protect applicant identity and any ensuing bias. Of the responses, 26% reported using LLM tools for minor edits in their personal statements, and no applicant reported the use of AI tools for major sections of their personal statements. It remains difficult to differentiate the use of AI tools for paragraph generation as opposed to minor editing tasks of the applicant's original work. As AI technologies continue to evolve, program directors should consider how LLM-assisted writing influences their ability to holistically assess an applicant and encourage more explicit guidelines for the use of these tools.

## Introduction

Amidst the growing popularity of generative artificial intelligence (AI) tools such as large language models (LLMs) within the medical field and neurosurgical specialty [1], there has been growing interest in the potential for these tools to impact key processes, such as research and medical education. The recent surge in research analyzing the effects of ChatGPT (OpenAI, San Francisco, California, United States) and other similar LLMs has raised questions about their role in residency applications, particularly in personal statements [2]. 

LLMs, unlike more rudimentary machine learning, such as a logistic regression model, are designed using the most sophisticated neural network architecture within the field of deep learning. Models are taught to extract words, punctuation, and even complex tertiary ideas such as tone and theme from the user’s text entries in order to generate a text output for the user. The technology of back propagation, multidimensional vectors, and tokenization might be poorly understood by most applicants and program directors, but the user-friendly interfaces of the most popular LLMs allow residency candidates to enter chat prompts and receive custom-tailored responses to their queries within seconds. While LLMs have been shown to be beneficial in both clinical and research aspects of neurosurgery [3], a growing concern is the potential for rising trainees to present LLM-generated content as entirely their own, particularly in settings like residency applications where faculty seek to assess qualitative traits.

An applicant’s personal statement has traditionally been considered by program directors to be key in evaluating communication skills and qualitative traits not found elsewhere on the application [4]. Within the field of neurosurgery specifically, the National Resident Matching Program (NRMP) Program Director Survey Results in 2022 identified 85% of program directors using holistic review to identify promising candidates, and 80% using holistic review to increase resident diversity and improve applicant program alignment [5]. Neurosurgical residency programs matriculate one to four applicants to train over a seven-year period, marking the importance of qualitative attributes in the evaluation of an applicant’s fit into the residency program and departmental culture. Given the personal statement’s central role in the holistic review process, the increasing use of LLMs raises concerns about their impact on evaluating applicants' qualitative attributes. To our knowledge, this study presents the first survey-based analysis of LLM use in personal statements among neurosurgical residency applicants in United States programs during the NRMP Match.

## Materials and methods

This was a study based on a survey administered at the University of Miami (UoM) and the Medical College of Wisconsin (MCW) to 85 neurosurgery residency applicant interviewees during the 2024-2025 application cycle, surveying their use of any LLMs during the writing of their personal statement submitted during the Electronic Residency Application Services (ERAS) application process. Applicants were invited to complete the survey following their interview, and all responses were recorded anonymously.

Applicants who did not reach the interview stage or who did not accept interview offers were not included in this study. All invited applicants were contacted directly via their preferred email address from their application information, and the survey was conducted through free Google Forms tools (Google LLC, Mountain View, California, United States). Responses were decoupled from identifying information by research staff to ensure no bias during the application evaluation process. 

The survey comprised of a single question: “In writing your personal statement for neurosurgery residency applications, please select from one of the following”, with three answer choice options: “I used ChatGPT or AI to write the majority of my personal statement”, “I used ChatGPT or AI only for minor edits/error checking”, or “I did not use ChatGPT or AI for any portion of my personal statement”.

The data were analyzed both within each institution and in aggregate. Due to the anonymity of the responses, potential duplicate submissions could not be removed for applicants who may have interviewed at both institutions. IRB approval was not required due to no inclusion of patient data and the anonymity of survey participants. Results were not evaluated until the conclusion of the interview and match cycle to further ensure removal of bias from applicant evaluation. 

## Results

**Figure 1:**
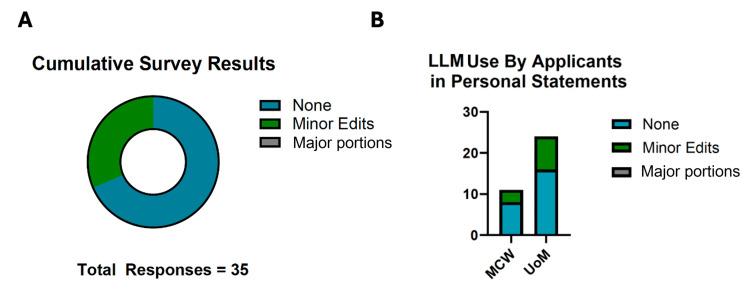
Use of artificial intelligence in writing personal statements (A) Pie chart of total survey responses. Responses showed 74% did not use an LLM in writing personal statements, while 26% consulted LLMs for minor edits. Notably, zero respondents indicated the use of LLMs for major portions of their personal statements. (B) Bar chart of responses per institution included in the analysis. MCW: Medical College of Wisconsin; UoM: University of Miami; LLM: large language model

A total of 85 students were invited to complete the survey after attending their residency interviews across both institutions. Of these, a total of 35 anonymous responses were recorded, yielding a 41% response rate. Any applicant who may have interviewed at both institutions and completed the survey twice could not be assessed due to anonymity. Aggregately, 0% of applicants reported using an LLM to write the major portions of the personal statements, 26% of applicants reported using AI for minor edits, and 74% denied the use of LLMs in forming personal statements. The results per institution are summarized in Figure 1. 

## Discussion

The proliferation of generative AI and public availability of LLMs has raised concern across many parts of medical education, including residency applications [6]. Despite the numerous benefits to readily available language tools and their time-saving advantages, LLMs are growing prevalent in the residency application process which are capable of true content generation. Given the results of our cross-sectional survey and the growing quality of LLMs over the past few years, we can reasonably expect LLMs to affect both an applicant’s approach to their submission and a program director’s evaluation of each application.

LLMs can be used to generate entire personal statements that are capable of passing as being written by a human [7-9], but the overarching consensus is that these wholly-generated essays are of lesser quality than organically-written text [10]. Nevertheless, students are not limited to a binary “all or nothing” essay-writing process; LLMs can be used to improve sentence structure, revise existing paragraphs, and make stylistic suggestions. Per the results of the survey, students who opt to use LLMs are choosing to use the tools in this fashion, with about a quarter of respondents indicating this use. Survey results cannot be verified, so the actual number of applicants involving AI in their personal statement may be lower or higher. Guidelines have been proposed by the Association of American Medical Colleges (AAMC) for how to utilize AI tools when writing residency applications, but the ability to police and enforce these guidelines remains nearly impossible [11].

In recent years, neurosurgery residency program directors have adapted their evaluation of candidates to the pass/fail grading system of the United States Medical Licensing Examination (USMLE) Step 1 exam [5], leaning further into Step 2 scores, research portfolios, and holistic review in choosing candidates. It is seen that the trend in medical education has shifted grading to pass/fail, quartile rankings, or an honors/high pass/pass marking system; the removal of traditional quantitative grades and class ranks has made qualitative evaluation more imperative than ever for understanding the readiness of each applicant for a residency position. The rise of LLMs threatens a program director’s ability to trust the qualitative insight into an applicant’s personal statement, depersonalizing the holistic review process.

These shifts away from quantitative assessment come in the context of recent literature exposing potential bias in faculty letters of recommendation (LORs), further complicating the review of applicants. A recent study shows that 58% of standardized neurosurgery LORs written by department chairs and 44% written by program directors rank students in the top 5% of students they have evaluated across all categories [12], raising more concern for the reliability of qualitative application material. Moreover, evidence from LLM analysis of LORs has suggested that the specific language used in the letter is predictive of an applicant matching into neurosurgery [13]. LLMs are capable of learning these trends and replicating language commonly found in successful letters or personal statements, potentially influencing a subsequent applicant’s submission. Currently, medical trainees are not educated in “prompt engineering”, or the art of customizing text inputs to tailor an LLM response; mastery of these LLM tools will only narrow the gap between applicant-written essays and AI-influenced prose [14]. It remains unclear how program directors will respond to these AI technologies, given the uptick in their use by applicants, or indeed what guidelines each program will set forth for acceptable use of LLMs during the application process.

The field of neurosurgery demands sharp residency application evaluation and due diligence during the screening of applicants, given the rigor of residency training. Incoming residents learn to manage large patient censuses, triage urgent consultations, master delicate surgical techniques, and perfect their anatomical knowledge base. Moreover, training positions are very limited, with only 241 positions across 116 residency programs in 2024 [15]. Those 241 positions received 423 applications, 299 coming from graduates of United States medical schools. Program directors rely on deciphering capability for teamwork, academic interest, work ethic, interpersonal skills, and communication style in selecting incoming residents in order to make informed rank-list decisions. Given that the use of ChatGPT has been disclosed by survey respondents in this study, personal statements remain at large risk for ChatGPT encroachment.

As quantitative metrics have changed significantly in recent years, the increasing use of LLMs introduces new challenges to the qualitative assessment of applicants, further complicating the residency selection process. Our findings demonstrate that a notable portion of neurosurgery applicants have integrated LLMs into their personal statement writing, primarily for minor edits and refinements rather than full content generation. While this suggests that applicants are using AI as an augmentative tool rather than a complete replacement for original writing, the evolving capabilities of LLMs raise concerns about the authenticity of qualitative prose. The ability of these models to mimic successful language patterns found in personal statements and letters of recommendation poses a potential risk to holistic review, as program directors may struggle to differentiate between an applicant’s true voice and AI-generated refinements. Governing bodies such as the ERAS committees and program directors have been called to action to set clear guidelines for residency applicant application essays [16-18] and residency applicant screening [19], turning to standardized disclosure systems or AI-detection software as potential next steps.

Limitations of the study

Our study is limited by the inability to verify survey responses. It is neither reasonable nor possible to confirm the extent of AI assistance in writing personal statements; thus, this investigation requires trust in the integrity of survey respondents. This limitation was mitigated through anonymity, but must be acknowledged. We would logically expect students to underreport rather than overstate the use of AI tools. Our study was also limited by its scope to two institutions and 41% response rate. All survey participation was voluntary, but we acknowledge the potential bias this introduces from a limited, non-random sample.

## Conclusions

As residency programs continue to refine their selection criteria in response to shifting grading systems, USMLE changes, and evolving application trends, the role of AI-assisted writing in residency applications will require ongoing scrutiny. Program directors and governing bodies are encouraged to outline and establish clear guidelines for the appropriate use of such tools within residency applications to ensure clarity from both applicants and institutions. Future discussions should focus on establishing clearer guidelines for AI use in medical education, ensuring transparency in the application process, and preserving the integrity of qualitative evaluations in neurosurgical residency selection.
